# Extravasation of catheter tip following central venous catheterisation: A near fatal complication

**DOI:** 10.4103/0019-5049.72651

**Published:** 2010

**Authors:** Mridu Paban Nath, Sachin Gupta, Anulekha Chakrabarty

**Affiliations:** Department of Anaesthesiology and Critical Care, Gauhati Medical College Hospital, Guwahati, Assam - 781 005, India

**Figure 1:**
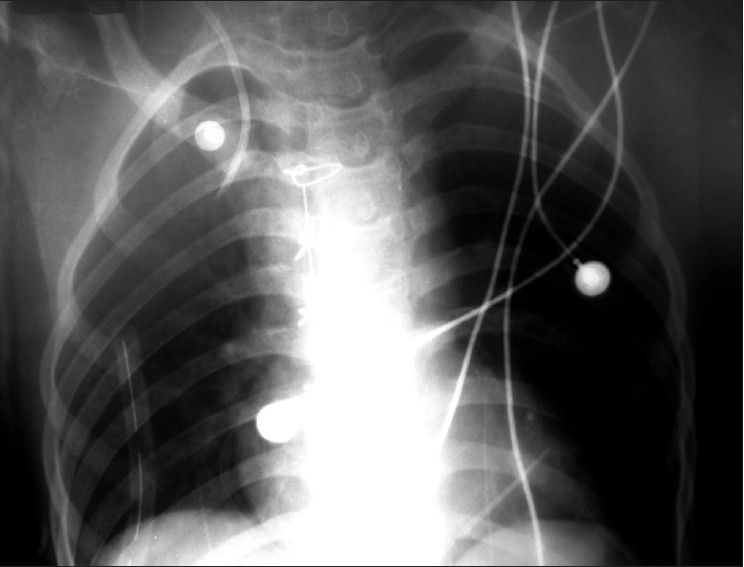
X-ray chest showing tip of the central venous catheter at apex of the right lung

Sir,

A 14-year-old patient underwent sub-aortic membrane resection with cardiopulmonary bypass support. Post induction, right internal jugular vein (IJV) cannulation was done with 16-G, 18-G, and 18-G triple lumen catheter in a single attempt. Intravascular placement of the catheter was confirmed after aspiration of blood from the proximal port and was connected to the transducer for central venous pressure (CVP) monitoring. The middle port was connected to the intravenous fluid and the distal one was connected to inotropes. The CVP measured low (3 mm Hg) and, wave form not being clear, it was assumed that the catheter tip might be stuck in the vessel wall. Intraoperatively, fluid was given through the peripheral line. Towards the end of the procedure, right pleural cavity was sucked and a chest drain was given. Surgery was completed uneventfully. Patient was shifted to the intensive care unit (ICU) and elective ventilation was started. Postoperatively, CVP was still on lower side (3 mm Hg) and 500 colloid (Hestar 6%) was infused through the middle port of the CVP line. Suddenly, the right chest drain started collecting with serous type collections and about 500 ml was collected over half an hour. The possibility of misdirected catheter tip at the pleural cavity was suspected, for which the whole colloid was drained out through the right-sided chest drain. Urgent chest X-ray confirmed the position of the catheter tip at the right pleural cavity [Figure 1]. Then, it was confirmed that half of the catheter was inside the vessel and half outside in the right pleural cavity, resulting in the erroneous CVP measurement.

Then, removal of the catheter was planned from the right side. As soon as the catheter was removed, BP went down to 50 mm Hg and the chest drain started collecting blood (400 ml). As an emergency, a 16-G cannula was inserted at left external jugular vein for rapid volume infusion. Bleeding from the torn end of the vessel was suspected after removal of the catheter. Urgent re-exploration was done, and intraoperatively, a rent in the lower end of the IJV was found, which was profusely bleeding through the apex of the right lung. Rent was repaired and about 3 l of blood was evacuated from the right pleural cavity. Once haemostasis was confirmed, blood transfusion was started and the BP reached 110 mm Hg.

Central venous catheters (CVC) are an essential component of modern critical care. Despite their utility, placement of CVCs is often associated with complications such as[1,2] malposition of the catheter and complications relating to perforation and/or injury of nearby blood vessels and structures.

Cannulating the left internal jugular vein has a higher chance of malposition because the left brachiocephalic vein has a more transverse lie, thus making the catheter more prone to angulation. There has also been a case reported of inadvertent placement of a jugular venous catheter into the left superior intercostal vein.[3]

Accidental puncture and perforation of blood vessels or injury to nearby structures usually results in a more catastrophic outcome and is not uncommon. In one study, carotid artery puncture occurred in 8.3% of patients undergoing internal jugular vein cannulation.[4] Schummer *et al*.[5] reported a case similar to our patient, with unrecognised stenosis of the superior vena cava (SVC). Perforation occurred in the SVC after catheterisation of the left internal jugular vein with a haemodialysis catheter. Extravascular positioning of the catheter was unrecognised and the patient subsequently died of complications.

Partial placement of catheter (half of the catheter inside the vessel and half outside) with distal part extravasation is seen very rarely, and till now, such a case has not been reported in the literature. In our case, the catheter up to the proximal port was partially inside the vessel, which we could aspirate, but rest of the catheter was outside the vessel and pierced the vessel wall, leading to migration in the right apical pleural cavity. Our diagnosis became late because the middle and the distal ports were not aspirated intraoperatively. Postoperatively, when the collection in the right chest drain became profuse after administering fluid through the middle port of the catheter, such a situation was suspected and later confirmed by radiography. A post-procedural chest radiograph is generally considered essential in identifying malposition of the catheter.

Once such vascular complications are diagnosed, the management becomes important. Our patient suddenly developed hypotension as he started bleeding profusely from the rent in the vessel after removing the catheter. Although re-exploration and surgical haemostasis was done, we realised that such chaotic situations could have been avoided by removing the catheter inside the operation theatre.

We recommend from this experience that free venous outflow must be carefully checked in all the ports of CVP catheter, and following placement of such catheter, chest radiograph should be completed to confirm the position. If such partial vessel extravasation is there, it should be removed in operation theatre with blood products ready in hand for resuscitation.
